# No Benefit in Memory Performance after Nocturnal Memory Reactivation Coupled with Theta-tACS

**DOI:** 10.3390/clockssleep6020015

**Published:** 2024-03-25

**Authors:** Sandrine Baselgia, Florian H. Kasten, Christoph S. Herrmann, Björn Rasch, Sven Paβmann

**Affiliations:** 1 Cognitive Biopsychology and Methods, Department of Psychology, Université de Fribourg, 1700 Fribourg, Switzerland; sandrine.baselgia@unifr.ch; 2 Centre de Recherche Cerveau & Cognition, CNRS & Université Toulouse III Paul Sabatier, 31062 Toulouse, France; florian.kasten@cnrs.fr; 3 Experimental Psychology Lab, Department of Psychology, Carl von Ossietzky Universität, 26129 Oldenburg, Germany; christoph.herrmann@uni-oldenburg.de; 4 Department of Neurology, University Medicine Greifswald, 17475 Greifswald, Germany

**Keywords:** memory, non-REM sleep, transcranial alternating current stimulation, targeted memory reactivation

## Abstract

Targeted memory reactivation (TMR) is an effective technique to enhance sleep-associated memory consolidation. The successful reactivation of memories by external reminder cues is typically accompanied by an event-related increase in theta oscillations, preceding better memory recall after sleep. However, it remains unclear whether the increase in theta oscillations is a causal factor or an epiphenomenon of successful TMR. Here, we used transcranial alternating current stimulation (tACS) to examine the causal role of theta oscillations for TMR during non-rapid eye movement (non-REM) sleep. Thirty-seven healthy participants learned Dutch–German word pairs before sleep. During non-REM sleep, we applied either theta-tACS or control-tACS (23 Hz) in blocks (9 min) in a randomised order, according to a within-subject design. One group of participants received tACS coupled with TMR time-locked two seconds after the reminder cue (time-locked group). Another group received tACS in a continuous manner while TMR cues were presented (continuous group). Contrary to our predictions, we observed no frequency-specific benefit of theta-tACS coupled with TMR during sleep on memory performance, neither for continuous nor time-locked stimulation. In fact, both stimulation protocols blocked the TMR-induced memory benefits during sleep, resulting in no memory enhancement by TMR in both the theta and control conditions. No frequency-specific effect was found on the power analyses of the electroencephalogram. We conclude that tACS might have an unspecific blocking effect on memory benefits typically observed after TMR during non-REM sleep.

## 1. Introduction

Sleep improves and consolidates memory in a variety of tasks [1,2,3]. It is widely assumed that the benefit of sleep on memory is supported by an ongoing hippocampal-neocortical dialogue enabling integration of newly learned memories into long-term memory stores [4,5]. According to the active system consolidation hypothesis, memory traces are spontaneously reactivated mainly during non-rapid eye movement (non-REM) sleep stages N2 and slow wave sleep (SWS) in both the hippocampus and the cortex. Slow oscillations (0.1–1 Hz) are believed to synchronise these reactivations as well as hippocampal sharp-wave ripples and thalamo-cortical spindles [3,6]. This synchronisation is assumed to optimise the hippocampal-neocortical dialogue during sleep needed for the formation and stabilisation of long-term memory traces [7].

Targeted memory reactivation (TMR) is an established method to elicit memory reactivations during sleep [8]. It consists of presenting sensory cues during sleep to activate associated memories from previous learning. The effects of the cueing are evaluated on post-sleep memory performance [2]. A recent meta-analysis including results from over 70 TMR studies concluded that TMR applied during N2 and SWS was selectively improving memory for the associated information [9].

When investigating TMR-elicited neural activity with EEG, successful reactivation of declarative memory during sleep is typically associated with event-related increases in theta oscillations (4–7 Hz) followed by an increase in spindle oscillations (11–15 Hz), which are more pronounced for later remembered vs. later forgotten items [10,11,12,13,14]. Several studies have shown effects in the theta rhythm after successful cueing [10,11,13,14,15], with the increase in induced theta power being typically reported 500 to 1200 ms after cue onset [10,11,13,14,15]. In a recent study, it was also suggested that theta oscillations (at 5 Hz) may play an important role in orchestrating the reactivation of information both during sleep and wakefulness [16]. During wake, activity in the theta range has been associated with successful memory processes [12,17,18,19], as shown with an increased activity both during the encoding and retrieval of later correctly remembered items compared to not remembered items [20,21,22]. Recent studies suggested that, during encoding, hippocampal theta activity allows the transfer of information from the hippocampal to the neocortical structures by coordinating the activity in both structures [7,23,24,25,26]. Therefore, theta oscillations improve the communication between the hippocampus and the neocortex [27] by creating time windows in which the information can be efficiently received and processed [28]. During non-REM sleep, theta activity is assumed to represent the reinstatement of the learned memory trace [12,13]. Given these findings associating theta activity to declarative memory processes, theta activity seems to play an important role in the consolidation and stabilisation of memory traces during non-REM sleep. However, as most evidence of the involvement of theta in successful reactivation and consolidation of memories during sleep is correlational, the functional relevance of theta in these processes remains unclear.

To investigate the functional relevance of a specific frequency band, transcranial alternating current stimulation (tACS) is a valid tool that allows to modulate endogenous neural oscillations [29]. By applying an alternating current to the scalp, tACS can be used to entrain and amplify intrinsic oscillations at the stimulated frequency [30,31] or enhance the synchronisation between brain regions [32]. Studies during wakefulness have shown that tACS can directly affect the behavioural performance in given cognitive processes, e.g., [29,32,33], which therefore allows to observe relationships and establish causal links between brain oscillatory activity and brain functioning [32,34]. Studies applying transcranial electrical stimulation during non-REM sleep have mostly used low frequencies (0.75 Hz) [35,36,37]. To our knowledge, only two studies have applied electrical stimulation during sleep in the theta range and examined memory processes: Marshall and colleagues [35,38] reported stimulation-induced decreases in slow oscillation power after theta-oscillating tDCS along with a decreased post-sleep declarative memory performance. However, those studies used transcranial direct current stimulation (tDCS) whose mechanism differs from tACS by inducing either depolarisation or hyperpolarisation [32]. TDCS thus directly affects the firing rate of neurons, whereas tACS regulates the firing rate of the neurons in an oscillatory manner without changing the average firing rate of neurons [33]. No study has investigated the combined effects of theta-tACS and TMR on declarative memory performance.

Therefore, in this study, we aimed at investigating the functional role of theta in sleep-associated memory processes by presenting reminder cues coupled with tACS in the theta frequency range. The effects of TMR coupled with theta-tACS were compared to cues coupled with a control stimulation, presented in the same night. In addition, we compared two different stimulation paradigms, where one group of participants received a continuous stimulation and another group received a time-locked stimulation targeting the time window where the increase in theta power has typically been reported [10,11,13,14,15]. The stimulation was designed to reach and synchronise the hippocampus and the prefrontal cortex in order to optimise the communication between the two regions. The Dutch–German vocabulary task used in this study had previously been used in our laboratory, e.g., [39] and has shown increased memory performance after reactivation during sleep [12,15]. We predicted that the coupling of theta-tACS to TMR cues during non-REM sleep would result in a memory-enhancing effect as compared to a control-tACS with both stimulation paradigms (continuous and time-locked). In addition, we expected a greater theta activity during the cueing of later remembered words compared to later forgotten words, with a greater effect for the cues coupled with theta-tACS.

## 2. Results

### 2.1. Effects of Verbal Cueing Coupled with tACS on Memory for Dutch Vocabulary

The main aim of this study was to investigate whether theta-tACS coupled with TMR could increase memory performance in two different stimulation paradigms (continuous and time-locked). In contrast to our hypothesis, neither continuous nor time-locked stimulation with theta-tACS shortly after/during the re-exposure of Dutch words increased memory performances tested after sleep: when Dutch words were reactivated during continuous theta-tACS stimulation in the continuous group, participants remembered 94.10 ± 2.34% of the Dutch words remembered before sleep (with pre-sleep memory performance set to 100%) compared with 98.35 ± 2.14% in the control stimulation condition (*t*_20_ = −1.04, *p* = 0.310, *d* = 0.31). When the words were reactivated followed by 2 s of theta-tACS in the time-locked group, participants remembered 94.37 ± 2.42% of the word pairs they remembered before sleep compared with 92.84 ± 1.76% in the control stimulation condition (*t*_15_ = 0.34, *p* = 0.738, *d* = 0.12). Thus, stimulation with theta-tACS either during or shortly after TMR of Dutch words did not improve memory consolidation compared with a control stimulation (tACS in the beta frequency range).

**Figure 1:**
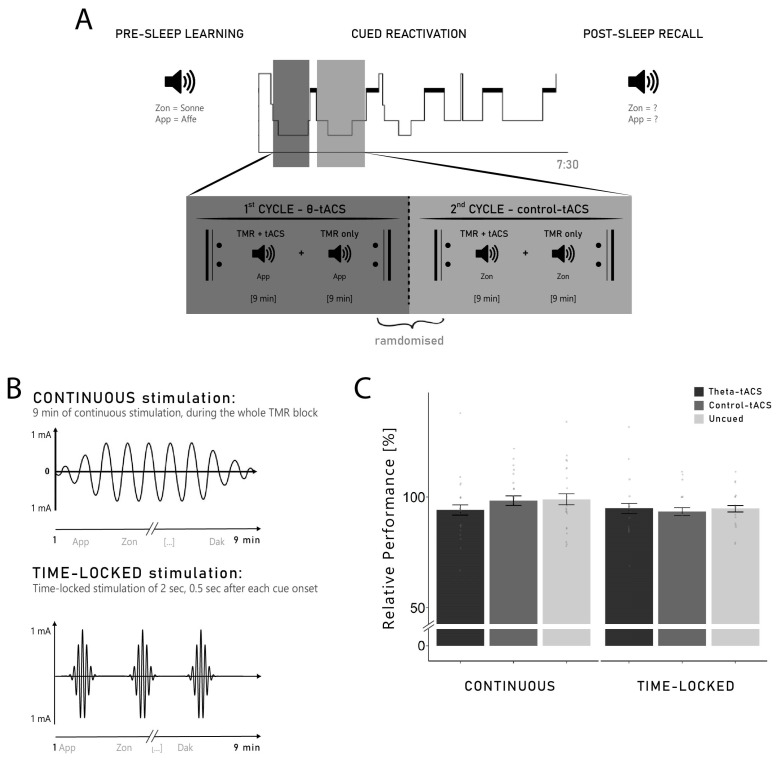
(**A**) **Experimental procedure**. Participants learned 140 Dutch–German word pairs before sleep. During subsequent non-REM sleep, 84 Dutch cues were presented again. The cueing of vocabulary occurred during the first two sleep cycles: in one cycle, the cues were coupled with theta-tACS, and in the other cycle, they were coupled with a control stimulation (in the beta frequency range). The order of the stimulation was randomised. One group of participants (*n* = 21) received a continuous stimulation, while the other group (*n* = 16) received a time-locked stimulation (0.5 s after each cue onset). In each cycle, we alternated TMR blocks coupled with stimulation with TMR blocks without stimulation until a maximum of 5 blocks or until REM sleep was noticed. In the morning, participants were tested on the German translation of the Dutch words using a cued recall procedure. (**B**) **Difference between continuous and time-locked stimulation**. The continuous stimulation was started at the beginning of the TMR blocks and lasted for its whole duration (9 min). The time-locked stimulation was a 2 s stimulation, applied 0.5 s after each TMR cue of the 9 min block. (**C**) **Relative difference between pre- and post-sleep recall**—with performance of the pre-sleep recall set to 100%—in the continuous and time-locked groups for TMR cues coupled with theta-tACS, TMR cues coupled with a control stimulation and unpresented words (uncued). Neither time-locked nor continuous stimulation with theta-tACS during the re-exposure of Dutch words increased memory performance tested after sleep. Thus, stimulation with theta-tACS either during or shortly after the TMR of Dutch words did not improve memory consolidation compared with a control stimulation and with unpresented words.

Moreover, the re-exposure of Dutch words generally did not improve memory for Dutch–German word pairs compared to words not presented during sleep: participants in the continuous group remembered 98.97 ± 2.51% of the uncued words, which again did not differ from reactivated words in the theta-tACS and control-tACS conditions (*F*_2,40_ = 0.70, *p* = 0.502, *η*^2^ = 0.03). Similarly, participants in the time-locked group remembered 94.26 ± 1.56% of the words not presented during sleep (uncued words), which did not significantly differ from both the theta-tACS and control-tACS words (*F*_2,30_ = 0.08, *p* = 0.921, *η*^2^ = 0.01). The overall ANOVA including the three levels of the stimulation factor (theta-tACS, control-tACS, uncued) and the group factor (continuous vs. time-locked) revealed no significant results (all *p*-values > 0.184; see Figure 1C). Thus, on the behavioural level, we were not able to replicate the well-known memory enhancing effect of verbal TMR—see [9]—in our study.

Our failure to replicate the TMR effect on memory was not related to differences at encoding before sleep: on average, participants correctly recalled 43.12 ± 2.19% of the 132 words (56.92 ± 2.89 words; range 22–101 words), indicating a medium task difficulty which is considered ideal for observing TMR effects. Performance in the pre-sleep recall corresponds to the results obtained in our previous studies, e.g., [10,11,39]. We did not observe any group differences in pre-sleep memory performance between the two experimental groups (factor group: *F*_1,35_ = 0.00, *p* = 0.966, *η*^2^ < 0.01), no difference between later cued with theta-tACS, later cued with control-tACS and uncued words (factor stimulation: *F*_2,70_ = 0.93, *p* = 0.399, *η*^2^ = 0.03) and no interaction between group and stimulation (interaction: *F*_1,35_ = 0.56, *p* = 0.571, *η*^2^ = 0.02; see Table 1 for descriptive statistics).

Additional analyses on words that were gained (i.e., Gains, words not correctly remembered before sleep, but correctly remembered after sleep) or lost (i.e., Losses, words correctly before sleep, but not correctly remembered after sleep) across sleep are provided in Supplementary Materials (see Table S1 and Figure S2).

### 2.2. Neural Correlates of Cueing with tACS

**Figure 2:**
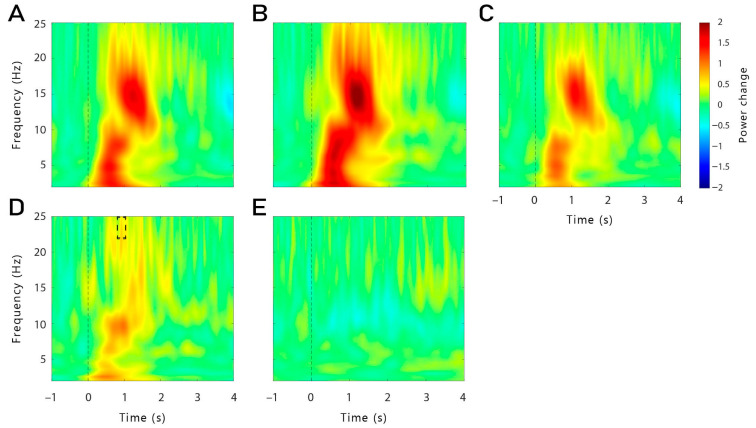
**Averaged oscillatory responses** to words presented during non-REM sleep recorded in all channels (F3, F4, P3, P4, F7, F8, Fz, FC5, FC6, CP1, CP2, CP5, CP6, Pz). (**A**) **Oscillatory power changes for all words (Hits, Misses) presented during non-REM sleep in both groups (continuous, time-locked)**. Word presentation during sleep led to the typical brain response encompassing an increase in the slow-wave activity (SWA), theta, and alpha bands (1–12 Hz), followed by an increase in the spindle and beta frequency bands (11–25 Hz). A similar pattern was observed in both groups individually, as shown in (**B**) for the continuous group and in (**C**) for the time-locked group. (**D**) **Comparison of the oscillatory power changes between the continuous and the time-locked group**: when comparing the general response between both groups, a higher beta power (21–25 Hz) was found in the continuous group, 0.89–1.09 s after cue onset, in the frontal regions (F3, F4, F7, F8, Fz, FC5, FC6; *p* = 0.028), as illustrated with the black dashed box. (**E**) **Comparison of the oscillatory power changes between Hits and Misses combined for all conditions and groups**: no difference was found.

Despite our failure to replicate the effects of TMR on memory on the behavioural level, we explored the oscillatory responses to words reactivated during sleep. In our first analysis, all artefact-free TMR trials of the TMR-only blocks (without stimulation) were included for both the continuous and the time-locked group. In general, word presentation during sleep led to the typical brain response encompassing an increase in the slow-wave activity (SWA), theta, and alpha bands (1–12 Hz), followed by an increase in the spindle and beta frequency bands (11–25 Hz, see Figure 2A). In both groups (continuous, time-locked), a similar pattern was observed (see Figure 2B,C), except that a higher beta power (21–25 Hz) was found in the continuous group, 0.89–1.09 s after cue onset in a frontal cluster of electrodes compared to the time-locked group (*p* = 0.028, see Figure 2D). To further investigate the oscillatory response to verbal cues on a neural basis, we first explored the general difference between event-related responses to reminder cues presented during non-REM sleep for subsequently remembered (Hits) and subsequently forgotten word pairs (Misses) for all groups and conditions combined. No significant clusters were found in this analysis (see Figure 2E).

**Figure 3:**
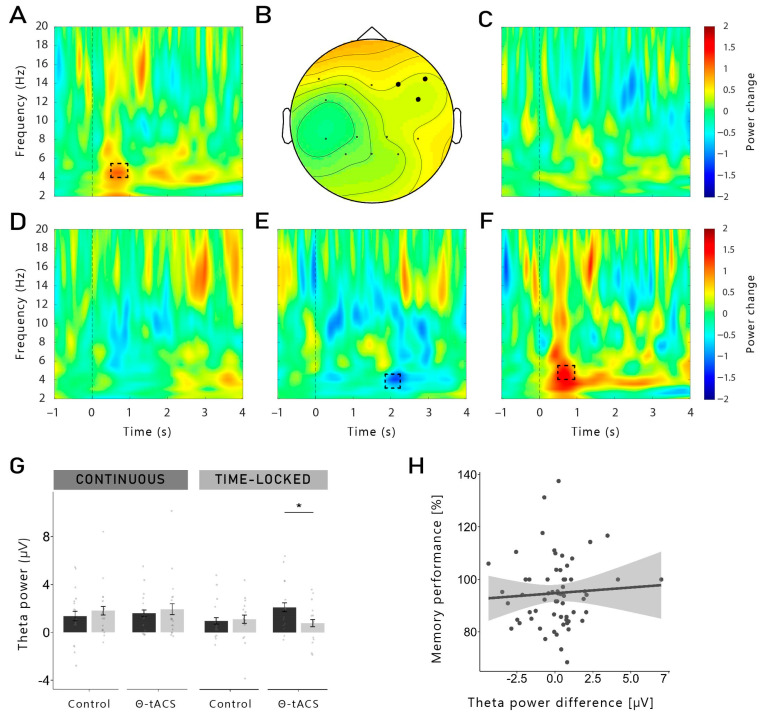
**Averaged oscillatory differences between Hits and Misses:** (**A**) In the time-locked group, a higher theta power (5 Hz) was observed for theta-Hits (compared to theta-Misses) 550–800 ms after stimulus onset (*p* = 0.044). The dashed box indicates the time (550–800 ms)-frequency (5–5.5 Hz) area used to illustrate the topographical distribution shown in subfigure (**B**). Significant electrodes are represented in filled black dots. (**C**) This cluster was found neither in the control-tACS of the time-locked group nor in the continuous group in both (**D**) the theta-tACS condition and (**E**) the control condition. (**E**) **Control-Hits vs. control-Misses in the continuous group**: there was a statistical trend for a lower theta power for Hits, 1.92–2.26 s after cue onset in the control-tACS condition, in the frontal region (*p* = 0.079). (**F**) **Theta-Hits vs. theta-Misses in the time-locked group, in the frontal right region**: the cluster shown in (**A**) was observed specifically in the frontal right region (F4, F8, FC6; *p* = 0.012) of the theta-tACS condition in the time-locked group. (**G**) **Mean theta power from the cluster shown in** (**F**): exploratory *t*-tests on the extracted mean theta power in this time window in the right frontal region revealed that theta power was indeed higher for Hits compared to Misses in the theta-tACS condition of the time-locked group (*t*_14_ = −2.71, *p* = 0.017, *d* = 0.70), but not in the continuous group, nor in the control-tACS condition (all *p*-value > 0.284). (**H**) **Correlation between theta power difference between Hits and Misses in the frontal right region and memory performance**, both groups and stimulation conditions together: no correlation was found. The shaded area represents 95% confidence interval. *: *p* < 0.05.

To investigate memory-dependent effects, we then analysed the differences between Hits and Misses separately for each group and for each stimulation condition (see Figure 3A–E). Since the changes in theta have previously been reported 500–800 ms after cue onset [10,11,13,14,15], we restricted our analysis to this time window. In the time-locked group, a significant cluster was found in the theta-tACS condition (see Figure 3A,B). A higher theta activity (5 Hz) was observed for theta-Hits (compared to theta-Misses) 550–800 ms after stimulus onset (*p* = 0.044). This cluster was found neither for the control-Hits (compared to control-Misses) of the time-locked group (Figure 3C), nor in the difference in both conditions in the continuous group (theta-Hits vs. theta-Misses, control-Hits vs. control-Misses; Figure 3D,E).

As topographically fine-grained analyses are recommended, e.g., [40], we performed separate analyses in the right and left frontal regions (i.e., F4, F8, FC6 and F3, F7, FC5)—our target stimulation region. We observed a significant positive cluster in the right frontal region in the theta-tACS condition of the time-locked group (*p* = 0.012; see Figure 3F): theta-activity was higher for later remembered vs. later forgotten words, partly replicating previous findings [12,14,15]. This difference was not observed in the control-tACS condition. We observed no significant effects in the left frontal region, nor in the continuous group. Exploratory *t*-tests on the extracted mean theta power in this time window in the right frontal region revealed that theta power was indeed higher for Hits compared to Misses in the theta-tACS condition of the time-locked group (*t*_14_ = −2.71, *p* = 0.017, *d* = 0.70), but not of the continuous group, nor in the control-tACS condition (all *p*-values > 0.284; see Figure 3G).

No clusters were found for the other analysed frequency bands (beta, spindle). We also compared the difference between Hits and Misses in the theta-tACS condition to the difference in the control-tACS condition for both groups separately. No clusters were found in both groups.

We also analysed a later time window for theta frequency: 1.8–2.5 s after cue onset. No cluster was found in the time-locked group. However, in the continuous group, there was a statistical trend for a negative cluster in the control-tACS condition: 1.92–2.26 s after cue onset at 4 Hz (*p* = 0.079) in the frontal region, theta power was lower for Hits compared to Misses (*t*_16_ = 2.67, *p* = 0.017, *d* = 0.89; see Figure 3E). This effect was not found in the theta-tACS condition, nor in the time-locked group (all *p*-values > 0.265).

### 2.3. Correlations between Frontal Right Theta Power during Cueing and Memory Performance

We further investigated whether the increase in memory-dependent theta power observed in the time-frequency analysis was correlated to the behavioural memory performance across sleep. For this analysis, we correlated the theta power differences between Hits and Misses in the frontal right region with the retention performance across sleep (with pre-sleep performance set to 100%). As shown in Figure 3H, the theta power difference did not correlate with the memory performance (*r* = 0.06, *p* = 0.62). In addition, when separating for groups and stimulation conditions, no correlation was observed (all *r*-values < 0.14; all *p*-values > 0.616).

### 2.4. Power Analysis during Cueing

**Figure 4:**
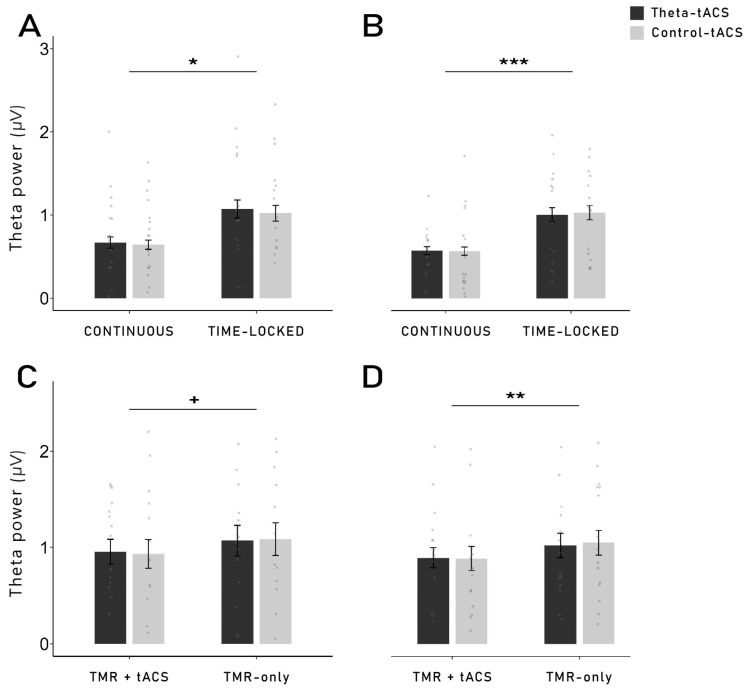
**Theta mean power during reactivation (TMR blocks without stimulation), 3–5 s after cue onset**: In the frontal left (**A**) and frontal right (**B**) regions, theta mean power was significantly higher in the time-locked group compared to the continuous group (left: *p* = 0.011; right: *p* = 0.001). Neither main effect of the stimulation condition nor interaction were observed. **Comparison of the theta mean power in TMR-only vs. TMR + tACS blocks in the time-locked group**: in the time-locked group only, an analysis was performed to compare the theta mean power in TMR blocks without stimulation (TMR-only) and TMR blocks coupled with stimulation (TMR + tACS), 3–5 s after cue onset. In the frontal left (**C**) and frontal right (**D**) regions, theta mean power in the time-locked group was higher in the blocks without stimulation compared to the blocks coupled with tACS (left: *p* = 0.057; right: *p* = 0.008). Neither main effect of the stimulation condition nor interaction were observed. +: *p* ≤ 0.06, *: *p* < 0.05, **: *p* ≤ 0.01, ***: *p* ≤ 0.001.

To further investigate general changes in oscillatory power after the stimulation, we performed exploratory power analyses 0–2 s (early) and 3–5 s (late) after each cue onset, in the theta (4–7 Hz) and beta (21–25 Hz) frequency range, in frontal left and frontal right regions. TACS produces heavy artefacts in the EEG which cannot be completely removed [41,42]. Due to such artefacts in the continuous group, we first analysed only the TMR blocks of both groups where no stimulation was applied (TMR-only; see Section 4.4 for details on the TMR procedure). In the late time window (3–5 s), theta power was higher in the time-locked group in both the frontal left and right regions (left: *F*_1,30_ = 7.27, *p* = 0.011, *η*^2^ = 0.20; right: *F*_1,30_ = 14.12, *p* = 0.001, *η*^2^ = 0.32; see Figure 4A,B) compared to the continuous group. In the beta frequency range, no difference between groups was found (both *p*-values > 0.106). Neither main effect of the stimulation condition nor interaction were observed (all *p*-values > 0.271). The same pattern was found in the early time window (see Table 2).

In the time-locked group, as the stimulation was only applied for 2 s, it was also possible to analyse the oscillatory power 3–5 s after the cues that were coupled with tACS (TMR + theta/control-tACS) compared to cues that were not coupled with tACS (TMR-only). In the frontal left region, theta mean power tended to be lower after the cues coupled with tACS (*F*_1,14_ = 4.28, *p* = 0.057, *η*^2^ = 0.23), while the difference was highly significant in the frontal right region (*F*_1,14_ = 9.57, *p* = 0.008, *η*^2^ = 0.41). However, neither differences were found between theta-tACS and control-tACS nor interaction (all *p*-values > 0.680; see Figure 4C,D). The beta frequency was impacted differently as a lower beta power was found in the frontal right for cues that were not coupled with tACS (TMR-only) in the theta-tACS condition, but the difference was not found in the control-tACS condition (interaction in the frontal right: *F*_1,14_ = 6.24, *p* = 0.026, *η*^2^ = 0.31). In the frontal left, however, only the main effect of the cues (TMR + tACS vs. TMR-only) was significant (*F*_1,14_ = 5.28, *p* = 0.037, *η*^2^ = 0.27), indicating that beta power was higher in the cues that were coupled to the stimulation, regardless of the stimulation condition (theta- or control-tACS; see Table 2).

### 2.5. Sleep

To control for sleep-dependent effects on memory performance, we performed general analyses on the sleep architecture and oscillatory power during non-REM sleep. For one participant of the time-locked group, the EEG sleep data were not recorded for the whole night; therefore, the analyses on general sleep parameters were performed on 36 participants. Overall, the participants spent an average of 502.49 ± 6.10 min in bed, with an average of 47.33 ± 1.28 min of stimulation. The participants of the time-locked group spent significantly more time in bed (520.33 ± 3.78 min) than the participants of the continuous group (489.74 ± 6.61 min; *t*_32.718_ = −2.93, *p* = 0.006, *d* = 0.91). Conversely, the stimulation time was significantly higher in the continuous group (49.69 ± 1.37 min) compared to the time-locked group (44.03 ± 0.93 min; *t*_33.955_ = 2.46, *p* = 0.019, *d* = 0.78). As time in bed differed in the two groups, we compared the percentage relative to the total sleep time for the time spent in each sleep stages. Participants of the time-locked group spent more time in slow-wave sleep (SWS; 16.49 ± 0.90%) and less time awake after sleep onset (WASO; 3.65 ± 0.37%) relatively to TST than participants of the continuous group (SWS: 12.80 ± 0.64%, *t*_23.179_ = −2.22, *p* = 0.036, *d* = 0.80; WASO: 6.28 ± 0.77%; *t*_30.803_ = 2.26, *p* = 0.031, *d* = 0.69). No difference was observed in the other sleep parameters (% of N1, % of N2, % of REM, sleep onset latency, SWS latency, REM latency; all *p*-values > 0.205; see Supplementary Table S2). Regarding these differences in sleep architecture, the time-locked group showed indicators of a better sleep quality than the continuous group. However, these differences in sleep did not influence memory performance in the expected direction: better sleep quality should result in better memory consolidation [43], independently of the stimulations, which was not observed in our data. The continuous group even showed a higher overall memory performance (mean percent difference: 96.57 ± 1.26%) than the time-locked group (93.22 ± 1.12%). The difference was not significant (*t*_34.108_ = 1.40, *p* = 0.169, *d* = 0.46). On the subjective level, the sleep quality score indicated a generally good sleep quality (3.43 ± 0.12). Participants in the continuous group showed a tendency towards a better subjective sleep quality (3.63 ± 0.11) than participants in the time-locked group (3.18 ± 0.12; *t*_30.886_ = −1.88, *p* = 0.070, *d* = 0.63).

We analysed the oscillatory power during non-REM sleep for theta and beta frequency bands as well as for the ratio between slow wave and beta activity (SWAB) in the frontal region. We first analysed the power in the whole night and then checked for differences in the first two sleep cycles (where the TMR and stimulation happened). Over the whole night, a higher power was observed in the time-locked group for theta (*t*_22.791_ = −2.67, *p* = 0.014, *d* = 0.93) and SWAB (*t*_25.735_ = −2.47, *p* = 0.021, *d* = 0.87) compared to the continuous group. No difference was found in the beta frequency band (*p* = 0.336). When looking at the first two sleep cycles separately, theta and beta power were higher in the time-locked group in the first cycle (theta: *t*_29.435_ = −2.66, *p* = 0.012, *d* = 0.90; beta: *t*_18.871_ = −2.25, *p* = 0.036, *d* = 0.83) compared to the continuous group. In the second cycle, the difference was observed only for theta (*t*_26.448_ = −2.40, *p* = 0.024, *d* = 0.83), but not for beta (*p* = 0.208). No differences were found for SWAB in both cycles (both *p*-values > 0.359). The change between cycle 1 and cycle 2 only approached significance in the beta frequency band (cycle × group interaction: *F*_1,35_ = 3.78, *p* = 0.060, *η*^2^ = 0.10; see Supplementary Table S2).

## 3. Discussion

In the present study, we investigated the effects of theta-tACS coupled with targeted memory reactivation (TMR) during sleep on post-sleep memory performance. Contrary to our expectations, TMR coupled with theta-tACS during sleep did not increase memory retention across sleep compared to an active control stimulation. Furthermore, we observed no beneficial effects of TMR on memory tested after sleep compared to words that were not reactivated during sleep. On the neurophysiological level, the reactivation of words during non-REM sleep led to typical responses with increases in the SWA, theta and alpha bands, followed by an increase in the spindle and beta frequency bands, indicating that the general brain response to reactivating verbal cues was not substantially altered by the stimulation. While we did not observe any memory-specific increase in theta when taking all conditions together, successfully remembered words that were coupled with time-locked theta-tACS showed a higher theta power compared to forgotten words, 550 to 800 ms after cue onset. This increase was more pronounced in the right frontal region. However, for both stimulation conditions, we did not find evidence for frequency-specific aftereffects, neither for theta nor for control stimulations.

Similarly to a recent study in our laboratory [39], we could not replicate the memory-enhancing effect of TMR shown in previous literature, e.g., [11,14]. While this failure questions the robustness and replicability of the memory benefits of verbal TMR during sleep, the coupling of TMR with tACS might have generally disrupted the possibility of reactivating memory in the current study. Moreover, while our time-locked stimulation was designed to target the time window where theta increases were observed in previous TMR studies (500–1000 ms after cue onset; e.g., [10,11,13,14,15]), it might have impaired the consolidation process by disturbing the following spindle activity. Indeed, two previous studies showed an impairment in memory consolidation when the presentation of the words was followed by another stimulus less than 1500 ms after [10,15]. This effect might be due to the suppression of the post-cue spindle activity needed for the integration and stabilisation of the new memory trace into the neocortical long-term network of pre-existing knowledge [11,13,44,45]. The time-locked stimulation used in this study—500 ms after the cue—might have similar detrimental effects on the necessary post-cue spindle activity. Antony and colleagues [46] also showed that the memory reactivation was most effective when the TMR was shortly followed by sleep spindle, emphasising that both neuronal oscillations are necessary for beneficial effects of TMR on post-sleep recall. In a similar study showing no benefits of TMR with the same task as the present study, Wilhelm and colleagues [13] also observed an increase in theta, but not spindle oscillations.

The idea of a stimulation-induced blocking of the TMR-induced memory reactivation process is supported by the results observed with the time-frequency analysis and power analysis during TMR. Indeed, we were expecting to observe a higher theta power after the cueing of subsequently remembered words compared to subsequently forgotten words. However, this result was observed only for words cued with time-locked theta-tACS, but not for continuous stimulation. It is important to note that no difference in theta power was observed during the pre-sleep recall (see Supplementary Figure S2). Therefore, our results cannot be explained by initial differences in the depth of encoding during learning. Moreover, theta power was even lower for subsequently remembered versus forgotten words in a later time window (1.9–2.3 s) in the continuous group. Therefore, it is possible that the continuous stimulation has long-lasting detrimental effects on the neurophysiological level during TMR-only blocks (which are the basis of this analysis, see Section 4.8) after the continuous stimulation blocks are over. In TMR-only blocks in the time-locked group, we observed the expected increased theta power for remembered words. Thus, long-lasting aftereffects in the time-locked stimulation seem to be unlikely. However, theta power was generally lower in the TMR + tACS blocks compared to the TMR-only blocks, possibly indicating direct detrimental effects of electrical stimulation on theta oscillations.

Independently of TMR, stimulation might generally impair the memory consolidation processes during sleep. Marshall and colleagues [38] used theta-tDCS during non-REM sleep and showed decreased post-sleep memory performances accompanied by a reduced spindle and slow oscillation activity during non-REM sleep. Based on stimulation studies during wakefulness, Murray and colleagues [47] suggested that theta-tACS applied over the temporal lobe during a memory task disrupted the communication between the temporal lobe and prefrontal cortex due to the interference of theta oscillatory activity. Therefore, it is possible that our stimulation paradigms (continuous and time-locked; theta-tACS and control-tACS) disrupted the consolidation processes naturally occurring during sleep.

Furthermore, the stimulation paradigms used in the present study might not have been optimal to reach our purpose. By stimulating both the hippocampus and the prefrontal cortex (in-phase: 0°; anti-phase: 180°), we aimed to synchronise the theta rhythm in both areas. Indeed, studies have highlighted the theta rhythm as a coordinator of the activity in the two regions allowing better transfer of information from the hippocampus to the cortical structures [7]. When analysing the TMR-only blocks (without stimulation), we did not observe any aftereffects of the stimulation on the frequency bands (theta, beta) in the region of interest (frontal). Therefore, it is possible that our stimulation did not have the expected effects (i.e., increase the power in the stimulated frequency). For our study, we virtually tested several different models of electrode placement and chose the parameters that were shown to best reach and bring the two structures in synchrony. Although it was recently shown that tACS applied on cortical areas can modulate hippocampal activity [48], it is possible that tACS might not be able to reach a deep structure such as the hippocampus. The hippocampal–neocortical communication that we were aiming to enhance might therefore not have been possible, preventing the improvement of memory consolidation. Moreover, the choice to stimulate both structures in-phase might also have impaired their communication. The transfer of information from the hippocampus to the neocortex might need a time difference that was not possible in our in-phase stimulation where we tried to perfectly synchronise the activity in both structures. Most wake studies that reported enhanced memory performance after theta-tACS applied the stimulation over parietal [49,50,51] or prefrontal cortex [48,52,53], but not over both regions simultaneously. Similarly to the present study, Alekseichuk and colleagues [54] administrated theta-tACS over the left prefrontal and left parietal cortices to synchronise both regions during a working memory task. Interestingly, their paradigm did not lead to a higher theta phase connectivity between the targeted regions, which, in turn, did not lead to a behavioural change in memory performance. While a stimulation on either the prefrontal or the parietal cortex might favour the communication between the two regions, it is possible that a simultaneous stimulation might impair this dialogue due to the lack of a time difference allowing the transfer of information. Furthermore, as electrical stimulation produces heavy artefacts in the EEG, it is hard to be certain that tACS does entrain the brain activity as expected [38]. Regarding the aftereffects of tACS, there is only mixed evidence of frequency-specific effects that might reflect cross-frequency interactions [31,55].

Our study included a rather small sample of healthy adults, which limits the generalisability of our results. Moreover, we used an active stimulation as a control condition to be able to detect frequency-specific effects. However, a sham stimulation might have provided valuable insights allowing for a comparison with an unimpacted consolidation process. Another limitation can be found in our design in which we decided to stimulate both frequencies in the same night, separating them in two different sleep cycles. Possible aftereffects from the first stimulation might influence the second stimulation, as previous studies showed behaviourally relevant aftereffects of tACS that lasted for over an hour e.g., [56]. Those aftereffects have nonetheless been minimised with the randomisation of the order of stimulation. However, with this design, it cannot be determined whether a specific stimulation condition was detrimental to the entire consolidation process or whether the succession of two different types of frequency stimulation in the same night was responsible for the lack of effects. Thus, future studies should try using two separate nights for the theta and the control stimulation, respectively. Furthermore, this design also led to fewer possibilities to reactivate each word. In the present study, it was possible to reactivate each word for a maximum of five times, while in previous TMR studies, it was typical to reactivate each word more than ten times, e.g., [11,14,39].

In conclusion, we were not able to replicate the memory-enhancing effect of TMR when coupled with theta-tACS. Therefore, our results do not allow to draw a clear conclusion on the functional relevance of theta in the reactivation and consolidation processes during sleep. According to recent studies, it is also possible that theta might not be related to memory consolidation but could be related to the processing of the sound cue itself [57]. In a recent study in our laboratory, an increase in theta oscillations following the cues was observed independently of any memory-enhancing effect of TMR [39]. This also suggests that an increase in theta oscillations might reflect successful reactivation rather than successful consolidation; see also [12]. Therefore, the topic remains of great importance and transcranial electrical stimulation can still be used to further investigate similar questions. However, more research is required to better understand the adequate stimulation parameters which can produce reliable improvements in cognition and future studies might consider comparing different stimulation modalities. Investigating this topic further could provide valuable insights into memory processes during sleep and is of particular importance in the context of memory-related disorders.

## 4. Materials and Methods

### 4.1. Participants

German-speaking participants (native speakers or bilinguals with at least a level of C2 proficiency in German) were recruited through advertisements placed at the University of Fribourg and on social media. They underwent a preliminary screening with questionnaires to assess exclusion criteria: current or past intake of medication having an impact on the central nervous system and on sleep (e.g., antidepressants, amphetamines), pregnancy, history of severe medical, neurological, psychiatric or sleep-related disorders, cognitive or hearing impairments, surgical operation in the past three months before the study, metallic implants on or in the head, head or brain injuries, history of epilepsy, and presence of scars on the areas of the stimulation electrodes. In addition to these exclusion criteria, depression was monitored by the German version of the Beck’s Depression Inventory-II (BDI-II; [58]) and any score above 14 on this questionnaire was excluded. None of the participants had shift work or intercontinental flights within six weeks prior to their participation in the study. They were instructed to avoid alcoholic and caffeinated beverages on experimental days as well as the day before.

Forty-three healthy German-speaking subjects completed both sessions of the experiment. We excluded five subjects from all analyses due to technical malfunctions or problems during the procedure. One subject was excluded due to an outlying score in the memory performance (>90% in pre-sleep recall). Thus, the final sample consisted of 37 subjects (28 women, mean age = 22.30 ± 3.26 [M ± SD], age range: 18–33 years). This sample size allows to detect a medium main effect and interaction of f = 0.25 (assumed correlation among repeated measures ρ = 0.5; standard 0.05 alpha error probability) with a power greater than 0.90. All participants received 130 CHF for their participation and provided written consent prior to their participation.

This study was approved by the Swiss Ethics Committee on research involving humans (Project-ID: 2018-02323). This study is part of a larger project examining the functional role of theta in the formation of declarative memories and was registered on OSF.io (https://osf.io/umhvg, accessed on 20 March 2024).

### 4.2. Design and Procedure

Each participant spent one adaptation night in the sleep laboratory of the University of Fribourg, which was followed by one experimental night, with at least two days (48 h) and at most one week in-between both sessions. Subjective sleep quality, sleep disturbances and chronotype were assessed using the German versions of the Pittsburgh Sleep Quality Index (PSQI; [59]), and the Morningness–Eveningness Questionnaire (d-MEQ; [60]). The mean on the total score of the PSQI—composed of seven indices measuring different aspects of sleep quality (e.g., subjective sleep onset latency)—was 3.81 ± 1.66 (M ± SD), which indicates that the sample was composed of good sleepers (a score higher than 5 indicates poor sleep quality; [59,61]). The Edinburgh Handedness Inventory was used to assess the right-hand dominance in everyday activities [62].

During the experimental night, each participant received both stimulations (theta- vs. control-tACS) in a randomised order. In addition to this within-subject factor, two separate groups of participants received different protocols of stimulation (between-subject factor ‘group’). One group of participants (*n* = 21) received a continuous tACS-stimulation applied during the whole duration of the cueing blocks (continuous group). For the other group of participants (*n* = 16), a 2 s stimulation was applied 0.5 s after each cue onset (time-locked group; see Section 4.4 for details). The two groups of participants were recruited sequentially.

The experimental session started at 7.00 p.m. with the attachment of electrodes for the stimulation and for recordings of electroencephalography (EEG), electromyography (EMG), electrooculography (EOG), and electrocardiography (ECG). Afterwards, participants had to perform a 3 min resting EEG with eyes closed. This resting EEG was used for the computation of the individual parameters for the stimulation. A training of the paired-associate learning (PAL) task was then performed by the participants in order to familiarise them with the vocabulary task (Dutch–German word pairs, see Section 4.3 for a detailed description of the task). Then, the participants had to complete two different lists of word pairs of the vocabulary learning task. The order in which the lists were presented was randomised across all participants and a short break was implemented in-between both lists. After completing both lists of the learning task, participants went to bed.

During N2 and SWS of the first two sleep cycles, the previously learned words were auditorily presented coupled with tACS. Participants received both tACS conditions in the experimental night (theta- and control-tACS): each of the two sleep cycles was assigned to one of the two stimulation conditions (see Section 4.4 for a detailed description of the reactivation procedure). We stimulated both frequencies in the same night because of the large inter-night variability in sleep architecture, e.g., [63,64] and memory performance, e.g., [65]. Using only one single night facilitates the direct comparison between the effects of the two stimulation conditions on oscillatory activity during sleep and memory processes. At 7.30 a.m., the participants were woken up and performed the recall of the previously learned word pairs (see Figure 1A for a summary of the experimental procedure). Before the tasks, participants indicated their general mood and well-being on the short German version of the Multidimensional Mood State Questionnaire (MDBF; [66]) as well as their level of wakefulness on a 10-point visual analogue scale (VAS). The VAS was again completed in-between the two learning lists and at the end of the second list. Subjective sleep quality was measured in the morning after the post-sleep recall with the sleep quality subscale of the SF-A/R [67], which consists of four indices assessing problems in the initiation and maintenance of sleep, early awakenings with inability to return to sleep, and general sleep characteristics. This sleep quality subscale assesses whether characteristics of good sleep quality are absent (score = 1) or strongly present (score = 5). At the end of the session, a post-stimulation questionnaire was completed to evaluate sensations related to the stimulation. This questionnaire was used to evaluate whether some effects of the stimulation might have been felt by the participants during sleep. The mean score on this questionnaire was 1.44 ± 0.24 (maximum score = 10), indicating that the stimulation did not lead to strong physical sensations during sleep. No difference was observed between the two experimental groups (continuous: 1.38 ± 0.23; time-locked: 1.53 ± 0.26; *t*_27.716_ = 0.31, *p* = 0.762, *d* = 0.11). Moreover, the score on this questionnaire was not correlated to the subjective sleep quality (*r* = −0.02, *p* = 0.90)

### 4.3. Vocabulary Task

The paired-associate learning (PAL) task used to assess declarative memory performance was retrieved from a study by Schreiner and Rasch [12]. It consisted of two lists of 70 Dutch words and their German translations each (see Supplementary Table S3). The lists were created to each contain an appropriate number of words chosen to be equal in length, to have an adequate level of difficulty as well as to be comparable in similarity measured by the Levensthein scale. The first two and the last two words of each list were used as buffer words to prevent primacy and recency effects and were discarded from analyses. A total of 132 word pairs were thus considered in the final analyses.

Each list was presented in three learning rounds in which the word pairs were randomly presented. The Dutch words and their German translations were presented acoustically (duration range: 400–650 ms) via loudspeakers (70 dB sound pressure level). In the first round, each Dutch word was followed by a fixation cross (500 ms) and subsequently by its German translation (intertrial interval: 2000–2200 ms). Participants were asked to memorise as many words as possible. In the second round, the Dutch words were presented again acoustically as a cue, followed by a fixation cross (500 ms). After listening to the Dutch word, the participants were asked to indicate whether they knew the words or not. If the word was known, they were instructed to vocalise the German translation. Afterwards, the correct German translation was presented again acoustically, irrespective of the given answer. In the third learning round, the cued recall procedure was repeated, without the feedback of the correct German translation. Here, the recall performance (pre-sleep recall) was used as pre-sleep learning performance. In this third round, participants correctly recalled, on average, 43.12 ± 2.19% of the 132 words (56.92 ± 2.89 words; range 22–101 words), indicating medium task difficulty without ceiling or floor effects. During the recall phase in the morning (post-sleep recall), the Dutch words from both learning lists were presented acoustically in a randomised order. In addition to the 140 words included in the pre-sleep learning lists, the 42 new words from the reactivation phase (see Section 4.4) were also presented. The recall followed the same procedure as the third round of the learning part (recall without feedback).

The tasks were performed on E-Prime, Version 2.0.10 and presented on a 24-inch screen. All verbal responses from the pre-sleep and the post-sleep recall were recorded via a voice recorder and stored for later analyses. The distance to the screen was maintained at 70 centimetres. As index of memory recall of the German translations, we calculated the relative difference between the number of correctly recalled words pre- and post-sleep, with the pre-sleep memory performance set to 100%: (post-sleep Hits × 100)/pre-sleep Hits.

### 4.4. Reactivation Coupled with tACS during Sleep

Single Dutch words were presented acoustically without their German translation during non-REM sleep in the first two sleep cycles (reactivation phase). The cues were presented via loudspeakers with the volume kept at 50 dB maximum and with 6000–8000 ms intervals. Of the 132 words learned before sleep, 84 were cued and 48 were not cued during sleep. An automatic MATLAB algorithm (Math Works, Natick, MA, USA) was used to create two different lists of word pairs to be cued coupled, respectively, with theta-tACS and control-tACS. Both lists were composed of 63 words each, with 21 words from each learning list and 21 new words serving as control stimuli. As specific effects of TMR were particularly shown on items that were ‘gained’ overnight (i.e., items not correctly remembered before sleep, but correctly remembered after sleep), e.g., [12,14,15], it was important to present both remembered and not remembered items during sleep. Therefore, the algorithm ensured an equal distribution of words that were remembered and words that were not remembered in the pre-sleep recall. This resulted in three word categories: words cued with theta-tACS (21 words from Learning List 1, 21 words from Learning List 2 and 21 new words), words cued with control-tACS (21 words from Learning List 1, 21 words from Learning List 2 and 21 new words) and uncued words (24 words from Learning List 1, 24 words from Learning List 2). Initially, we intended to reactivate 66 words with each stimulation. However, due to technical problems, only 63 words were reactivated. As this resulted in an unequal number between uncued words (48 words) and cued words (42 words for each stimulation), we randomly removed 6 uncued words for each participant to have an even distribution in the analysis.

Targeted memory reactivation (TMR) was coupled with tACS and applied during N2 and SWS of the first two sleep cycles where each cycle corresponded to one stimulation. The order of the stimulation was randomly assigned for each participant (e.g., theta-tACS in Cycle 1 and control-tACS in Cycle 2). TMR was administered in blocks of approximately 9 min, either coupled with tACS (TMR + tACS) or without tACS (TMR-only). As soon as the participants were in stable N2 sleep (approximately four minutes after the first signs of N2), we started the TMR procedure with a TMR + tACS block. After the reactivation of the 63 words coupled with tACS of the first block, a second block of the same 63 words was presented but without the stimulation (TMR-only). The alternating pattern of TMR + tACS and TMR-only was continued until a maximum of five blocks was reached or until the participants reached REM indicating the end of the first sleep cycle. If no sign of REM was visible, the first sleep cycle was considered over after 120 min of sleep. As each Dutch word was presented once per reactivation block, this resulted in a maximum of five exposure per word. At the end of the first sleep cycle, the same procedure was repeated during the second sleep cycle with the other reactivation list coupled with the other frequency of stimulation. During the reactivation, sleep was closely monitored by the experimenter and reactivation was paused whenever the participants woke up or showed any sign of REM.

### 4.5. Transcranial Alternating Current Stimulation (tACS)

Stimulation parameters used in this study were designed based on safety criteria [68] and on a montage modelled with ROAST (Realistic, vOlumetric Approach to Simulate Transcranial electric stimulation), Version 2.7.1 from MATLAB 2018b (The MathWorks, Natick, MA, USA). The models presented in Supplementary Figure S3 indicate that the stimulation parameters likely engaged our regions of interest (i.e., prefrontal cortex and hippocampus). The stimulation was applied by a battery-driven stimulator (DC-Stimulator; NeuroConn, Ilmenau, Germany) through four 5 × 5 cm conductive rubber electrodes—two target electrodes and two return electrodes. The two target electrodes were applied on EEG electrode sites FP1 and P7; the two return electrodes over sites FP2 and P8, based on the international 10–20 EEG system. This resulted in a 0° phase difference within each hemisphere (in-phase; [33]) and a 180° phase difference between both hemispheres (anti-phase). The current delivered by one battery-driven device was split between the target and return electrodes using a splitterbox (Medizin Technik Berger, Oldsloe, Germany). The electrodes were attached to the scalp using Ten20 conductive Neurodiagnostic Electrode Paste (Weaver and Company, Aurora, CO, USA). This montage was chosen as it was demonstrated to be the best in reaching the hippocampus and the prefrontal cortex and in achieving a synchronisation between these two regions. The impedance of the stimulation electrodes was kept below 5 kΩ (1.19 ± 0.21 [M ± SE]), and peak-to-peak intensity was 2 mA. With recent advances in stimulation techniques, temporal interference stimulation would have been an ideal option with greater depth of stimulation [69]. However, at the time when the study was designed, this technique was not established enough to be considered a valid option.

Each participant received both theta-tACS and control stimulation (beta-tACS): theta-tACS was applied during one of the first two sleep cycles and the control stimulation in the other cycle. The order of the stimulation was randomised (19 participants received theta-tACS in the first sleep cycle). For the stimulation of theta, we calculated the individual theta frequency (ITF) for each participant with the EEGLAB toolbox running on MATLAB 2018b. Based on previous studies [20,50,70,71], 5 Hz was subtracted from the individual alpha peak measured during the 3 min resting EEG recorded in the evening. When no alpha peak was detected, the fixed value of 5 Hz was used. For the control condition, we used the fixed value of 23 Hz, targeting beta frequency. The beta frequency was selected for the control condition as this frequency has not shown any consistent relationship to declarative memory performance and to the cortical–hippocampal interaction [17].

For both groups (continuous and time-locked), the stimulation was applied during the TMR blocks of approximately 9 minutes (see Section 4.4), but two different stimulation protocols were used for the different groups. In the continuous group, the stimulation was started at the beginning of the TMR block and was applied continuously for the entire duration of the block (9 min, with 10s ramp in/out). In the time-locked group, a 2 s stimulation was applied (no ramp in/out), 500 ms after each TMR cue (see Figure 1B). Therefore, the stimulation was applied 500–2500 ms after each TMR cue onset, as an increase in theta has previously been reported in this time window [10,11,13,14,15].

### 4.6. EEG Recording

Electroencephalographic (EEG) data was recorded with BrainVision Recorder 1.21 and a Brain Amp amplifier (Brain Products, Gilching, Germany) at a sampling rate of 500 Hz. We used Easycap Nets (Easycap GmbH, Herrsching, Germany) with either 32 (time-locked group) or 29 channels (continuous group). The used channels were the following, based on the international 10–20 system: Fp1, Fp2, FT9, FT10, F3, F4, C3, C4, P3, P4, O1, O2, F7, F8, T7, T8, P7, P8, Fpz, AFz, Fz, Cz, CPz, Pz, FC1, FC2, FC5, FC6, CP1, CP2, CP5, CP6, M1, and M2. FCz was used as a physical reference and AFz as a ground electrode. Two electrodes were used to collect electrooculographic (EOG) data, three chin electrodes were used to collect electromyographic (EMG) data, and three electrodes were used for electrocardiographic (ECG) data. Impedances were kept below 10 kΩ.

Following the AASM guidelines [72], electrodes were re-referenced to the mastoids for offline analysis and sleep scoring. Data was pre-processed with BrainVision Analyzer 2.2 (Brain Products, Gilching, Germany) using standard filter settings suggested by the AASM (second-order high- (0.3 Hz) and low-pass (35 Hz) filter; notch filter at 50 Hz). Sleep was scored offline by two independent raters according to standard criteria [72]; in case of disagreement, a third expert scorer was consulted.

### 4.7. Preprocessing and Artefact Rejection

Offline EEG pre-processing was performed using BrainVision Analyzer 2.2 (Brain Products, Gilching, Germany). Data was filtered using a second-order high- (0.1 Hz) and low-pass (40 Hz) filter with an additional notch filter at 50 Hz and re-referenced to averaged mastoids. The EEG data was segmented into 11 s segments, beginning 3 s before the stimulus onset. Segments were categorised based on the stimulation and performance in the post-sleep recall resulting in the following four categories: theta-Hits (i.e., words cued with theta-tACS and correctly remembered), control-Hits (i.e., words cued with control-tACS and correctly remembered), theta-Misses (i.e., words cued with theta-tACS and not correctly remembered) and control-Misses (i.e., words cued with control-tACS and not correctly remembered). Segments with artefacts were automatically rejected, and the following criteria were used to select the segments to keep: (1) a maximum difference in EMG activity <150 μV in both EMG channels, (2) a maximum voltage step in all EEG channels <50 μV/ms, (3) a maximum difference in EEG activity <500 μV in all EEG channels, and (4) a minimum activity in EEG activity >0.5 μV in all EEG channels. The number of removed segments was manually checked for each artefact rejection step. Automatic artefact rejection was chosen to have standardised criteria for all data sets. Each data set was additionally visually checked to ensure the quality of the artefact rejection.

Due to bad EEG quality and heavy artefacts, the data from five participants were excluded from further EEG analyses, resulting in a sample of 15 participants in the time-locked group and 17 participants in the continuous group for the time-frequency analyses and power analyses.

### 4.8. Time-Frequency Analysis

For the analysis of changes in event-related oscillatory power (time-frequency analyses), we used the Fieldtrip toolbox version 20210807 [73] running on MATLAB 2018b (Mathworks, Natick, MA, USA). Baseline normalisation was applied with a baseline period of −1 to 0 s before the stimulus onset. Data was then averaged per subject and per category (theta-Hits, theta-Misses, control-Hits, control-Misses; see Section 4.7), and grand averages of all categories were computed. A continuous wavelet transformation (complex Morlet wavelets, seven cycles) was performed on single trials (−3 to 7 s) to obtain the oscillatory power of frequencies between 0.5 to 30 Hz in steps of 0.5 Hz and 10 ms. Data included only TMR blocks without stimulation.

In the first analysis, we compared averaged Hits with averaged Misses separately for each stimulation condition (theta-tACS and control-tACS) and for each group (continuous and time-locked). Second, we computed the interaction by contrasting theta-Hits (Theta-Misses) with control-Hits (Control-Misses) separately for each group. As we were interested in differences in the theta frequency at the stimulation sites, we analysed this frequency band (4–7 Hz) in the time window 500 to 800 ms after cue onset across the frontal channels (F3, F4, Fz, F7, F8, FC5, FC6). We also analysed the differences in each hemisphere separately (frontal left: F3, F7, FC5; frontal right: F4, F8, FC6).

Exploratorily, we also analysed a later time window for theta frequency (1.8–2.5 s). As the control stimulation was applied in the beta frequency, we also explored differences in that frequency band (21–25 Hz; 0–6 s), as well as in slow spindles (11–13 Hz; 0.5–1 s) and fast spindles frequency band (13–15 Hz; 0.5–1 s). For all the time-frequency analyses, only the data from the TMR-only blocks were used (see Section 4.4).

Changes in event-related oscillatory power were also exploratorily investigated during the pre-sleep recall of the learning phase. We compared averaged Hits (subsequently remembered) with averaged Misses (subsequently forgotten) in the time-locked group only as important triggers were misplaced or missing in the continuous group making the analysis in this group unreliable. We analysed the theta frequency band in the time window from 500 to 1000 ms after cue onset across the frontal channels. The time window was chosen based on previous literature [20,21,22].

### 4.9. Power Analyses

To explore the changes in oscillatory power following the stimulation, we performed power analyses on two different time windows: (1) 0–2 s after the stimulus onset (early) and (2) 3–5 s after the stimulus onset (late). For the continuous group, only the data from the reactivation blocks without tACS were used (TMR-only) due to heavy artefacts caused by the stimulation. In the time-locked group, data from both the reactivation blocks with tACS (TMR + tACS) and without tACS (TMR-only) were used and analysed separately. Due to the stimulation, this was possible only for the late time window (3–5 s) in the time-locked group. After segmentation, the same artefact rejection procedure as for the time-frequency analysis was performed (see Section 4.7).

We used a fast Fourier transformation (Hanning Window tapering the endpoints to zero (10% Hanning Window), 0.25 Hz resolution, no padding) to investigate power differences during cueing and during sleep. Mean power values (μV) were exported for theta activity (4–7 Hz) and beta activity (21–25 Hz) in the EEG channels of interest (F3, F4, Fz, F7, F8, FC5, FC6).

We also performed an exploratory analysis to investigate the changes in oscillatory power during the tACS-free epochs of non-REM sleep in the first two sleep cycles separately and in the whole night. There, data was segmented in 30 s epochs of non-REM sleep based on sleep scoring results. Afterwards, data was segmented into equally sized segments of 2048 data points (4.096 s) with 102 points overlapping. Again, the same artefact rejection procedure was performed (see Section 4.7). We used the SleepTrip toolbox [74] for Matlab (Mathworks, Natick, MA, USA) to calculate the average power values (μV2) for theta (4–7 Hz) and beta (21–25 Hz) frequency bands in the main frontal EEG channels (F3, F4, Fz, F7, F8, FC5, FC6).

Power values exceeding mean activity of all channels by four standard deviations were replaced by the mean power separately for each subject. Next, power was averaged over the frontal region based on topography.

### 4.10. Statistical Analyses

Statistical analyses were performed using Rstudio version 2022.12.0 [75]. Data are presented as means ± standard error. The behavioural and power data were analysed with a repeated-measures analysis of variance (ANOVA) containing the within-subject factor ‘stimulation’ (theta-tACS, control-tACS, uncued) and ‘group’ (time-locked, continuous). We used a repeated measures ANOVA with ‘subjects’ as a random factor. Post hoc tests for significant interactions and main effects consisted of uncorrected paired Students’s *t*-tests. Exploratory analyses on other frequency bands, differences between hemispheres, and sleep parameters were not corrected for multiple comparisons. In the case of statistically significant results, effect sizes are reported with partial eta squared (*η*^2^) for main effects and interactions and Cohen’s *d* for *t*-tests. Associations were explored using Pearson correlations. The level of significance was set to *p* < 0.05.

Results of time-frequency analyses were compared using cluster-based permutation tests for dependent samples as implemented in the FieldTrip toolbox version 20210807 [73]. The maximum sum of *t*-values within every cluster served as the cluster-level statistic. Cluster-level alpha was set to 0.05. To consider the multiple comparisons problem, the cluster-level statistic was always calculated over 1000 randomly drawn data partitions. The proportion of random partitions exceeding the actually observed test statistic was calculated, resulting in a Monte Carlo *p*-value. The alpha level was set to 0.05 and corrected for two-sided testing. The alpha level was distributed over both tails by multiplying the probability with a factor of two, prior to thresholding it with the alpha level.

## Figures and Tables

**Table 1:** Percentages of correctly remembered words in pre-sleep and post-sleep recall in continuous and time-locked group for each stimulation condition (theta-tACS, control-tACS and uncued).

	Continuous			Time-Locked			Main Effect of Stimulation			Interaction Effect		
	Theta-tACS	Control-tACS	Uncued	Theta-tACS	Control-tACS	Uncued	*F*	*p*	*η* ^2^	*F*	*p*	*η* ^2^
Remembered words [%]												
Pre-sleep recall	45.69 ± 2.57	43.99 ± 2.67	45.58 ± 3.10	47.62 ± 2.16	44.49 ± 2.54	43.75 ± 1.67	0.93	0.399	0.03	0.56	0.571	0.02
Post-sleep recall	42.86 ± 2.67	43.08 ± 2.71	44.78 ± 2.99	44.20 ± 1.86	40.48 ± 2.01	41.37 ± 1.81	0.48	0.619	0.01	1.14	0.326	0.03
Percent difference	94.10 ± 2.34	98.35 ± 2.14	98.97 ± 2.51	94.37 ± 2.42	92.84 ± 1.76	94.26 ± 1.56	0.29	0.752	0.01	0.49	0.613	0.01

Notes: The percent difference indicates the percentage of words from the pre-sleep recall that were also recalled in the post-sleep recall, with pre-sleep memory performance set to 100%. Values are Means (M) ± Standard Error of Mean (SEM). *η*^2^ represents the effect sizes where a value below or equal to 0.02 reflects a small effect, a value between 0.02 and 0.14 reflects a medium effect, and a value above 0.14 represents a large effect.

**Table 2:** Power values during reactivation blocks.

	Continuous		Time-Locked		Main Effect of Group			Main Effect of Stimulation			Interaction Effect		
	Theta-tACS	Control-tACS	Theta-tACS	Control-tACS	*F*	*p*	*η* ^2^	*F*	*p*	*η* ^2^	*F*	*p*	*η* ^2^
Oscillatory Power [µV] in frontal left region TMR blocks without stimulation													
Theta, 0–2 s after cue	0.845 ± 0.090	0.860 ± 0.084	1.106 ± 0.078	1.164 ± 0.102	3.12	0.087 ^a^	0.09	0.20	0.661	0.01	0.07	0.789	<0.01
Theta, 3–5 s after cue	0.669 ± 0.067	0.642 ± 0.055	1.071 ± 0.109	1.023 ± 0.095	7.27	0.011 *	0.20	0.21	0.649	0.01	0.02	0.893	<0.01
Beta, 0–2 s after cue	0.007 ± 0.001	0.008 ± 0.001	0.010 ± 0.001	0.009 ± 0.001	2.27	0.142	0.07	0.01	0.930	<0.01	1.12	0.299	0.04
Beta, 3–5 s after cue	0.006 ± 0.001	0.007 ± 0.001	0.009 ± 0.001	0.009 ± 0.001	2.26	0.143	0.07	0.40	0.533	0.01	1.15	0.293	0.04
Oscillatory Power [µV] in frontal right region TMR blocks without stimulation													
Theta, 0–2 s after cue	0.748 ± 0.069	0.775 ± 0.073	1.165 ± 0.082	1.110 ± 0.072	7.91	0.009 **	0.21	0.03	0.858	<0.01	0.43	0.515	0.01
Theta, 3–5 s after cue	0.583 ± 0.049	0.578 ± 0.051	1.021 ± 0.086	1.046 ± 0.087	14.12	0.001 ***	0.32	0.02	0.902	<0.01	0.05	0.826	<0.01
Beta, 0–2 s after cue	0.006 ± 0.001	0.008 ± 0.001	0.010 ± 0.001	0.009 ± 0.001	2.46	0.127	0.08	0.00	0.976	<0.01	1.67	0.206	0.05
Beta, 3–5 s after cue	0.006 ± 0.001	0.007 ± 0.001	0.008 ± 0.001	0.009 ± 0.001	2.78	0.106	0.08	0.13	0.722	<0.01	1.26	0.271	0.04
Oscillatory Power [µV] in frontal left region TMR blocks coupled with tACS					Main effect of condition			Main effect of stimulation			Interaction effect		
Theta, 3–5 s after cue	-	-	0.956 ± 0.128	0.934 ± 0.148	4.28	0.057 ^a^	0.23	0.00	0.983	<0.01	0.18	0.680	0.01
Beta, 3–5 s after cue	-	-	0.009 ± 0.001	0.009 ± 0.001	5.28	0.037 *	0.27	0.21	0.655	0.01	2.24	0.157	0.14
Oscillatory Power [µV] in frontal right regionTMR blocks coupled with tACS													
Theta, 3–5 s after cue	-	-	0.894 ± 0.103	0.887 ± 0.125	9.57	0.008 **	0.41	0.01	0.929	<0.01	0.12	0.735	0.01
Beta, 3–5 s after cue	-	-	0.011 ± 0.002	0.008 ± 0.001	2.85	0.114	0.17	3.51	0.082 ^a^	0.20	6.24	0.026 *	0.31

Notes: Values are Means (M) ± Standard Error of the Mean (SEM). * indicates *p* < 0.05, ** indicates *p* ≤ 0.01, *** indicates *p* ≤ 0.001, ^a^ indicates *p* ≤ 0.09. Significant results are highlighted in bold. *η*^2^ represents the effect sizes where a value below or equal to 0.02 reflects a small effect, a value between 0.02 and 0.14 reflects a medium effect, and a value above 0.14 represents a large effect.

## Data Availability

The participants of this study did not give written consent for their data to be shared publicly, so due to the sensitive nature of the research supporting data is not available.

## Supplementary Materials

The following supporting information can be downloaded at: https://www.mdpi.com/article/10.3390/clockssleep6020015/s1, Table S1 and Figure S1: Number of Gains and Losses in continuous and time-locked group for each stimulation condition (theta-tACS, control-tACS and uncued); Table S2: Values and analyses of objective sleep parameters and oscillatory power during sleep; Figure S2: Average oscillatory power during the learning phase (pre-sleep recall) of the time-locked group; Table S3: Dutch–German word pairs; Figure S3: Models with ROAST.
