# Supplementary material for: No Benefit in Memory Performance after Nocturnal Memory Reactivation Coupled with Theta-tACS
Source: Clocks Sleep. 2024 Mar 25;6(2):211–33. doi: 10.3390/clockssleep6020015 (PMC11036246; doi:10.3390/clockssleep6020015)

Supplementary Material

for

No benefit in memory performance after nocturnal memory reactivation coupled with theta-tACS

Sandrine Baselgia[1], Björn Rasch[1*], Florian H. Kasten[2,3], Christoph S. Herrmann[4], Sven Paßmann[1,5,*]

[1] Cognitive Biopsychology and Methods, Department of Psychology, Université de Fribourg, Fribourg, Switzerland
[2] Centre de Recherche Cerveau & Cognition, CNRS, Toulouse, France
[3] Université Toulouse III Paul Sabatier, Toulouse, France
[4] Experimental Psychology Lab, Department of Psychology, Carl von Ossietzky Universität, Oldenburg, Germany
[5] Department of Neurology, University Medicine Greifswald, Greifswald, Germany

*Corresponding author:
Sven Paßmann, University of Fribourg, Department of Biopsychology and Methods, Rue P.-A.-de-Faucigny 2, 1700 Fribourg, Switzerland

e-mail: sven.passmann@unifr.ch

or

Björn Rasch, University of Fribourg, Department of Biopsychology and Methods, Rue P.-A.-de-Faucigny 2, 1700 Fribourg, Switzerland

Tel. +41 26 300 7637, e-mail: bjoern.rasch@unifr.ch

Table S1: **The two lists of Dutch-German word-pairs** used in the paired-associate learning (PAL) task, and the unlearned new words presented only during reactivation.

**List 1**		**List 2**		**New Words**
Been	Bein	Bij	Biene	Beek
Deur	Tuer	Kok	Koch	Beurs
App	Affe	Bloem	Blume	Brug
Baan	Beruf	Bloes	Bluse	Buis
Bel	Klingel	Boek	Buch	Deun
Blik	Blech	Boer	Bauer	Dolk
Bol	Kugel	Bot	Knochen	Eed
Bord	Teller	Bout	Bolzen	Fles
Borst	Brust	Buks	Buechse	Gids
Bos	Wald	Deel	Teil	Inkt
Breuk	Bruch	Gat	Loch	Keus
Buik	Bauch	Gif	Gift	Kroeg
Dak	Dach	Heup	Huefte	Kus
Deuk	Delle	Hoed	Hut	Lui
Dief	Dieb	Hut	Huette	Mist
Dijk	Deich	Jas	Jacke	Muts
Doek	Tuch	Kluis	Tresor	Muur
Drop	Lakritz	Krat	Kasten	Pad
Feit	Tatsache	Lijf	Leib	Pak
Fout	Fehler	Lip	Lippe	Peil
Geur	Geruch	Mand	Korb	Plas
Hak	Absatz	Mes	Messer	Plein
Hei	Heide	Mop	Witz	Pomp
Hiel	Ferse	Mug	Muecke	Prei
Hok	Schuppen	Munt	Muenze	Prent
Hout	Holz	Neef	Neffe	Prijs
Hulp	Hilfe	Oog	Auge	Reis
Ijs	Eis	Pan	Topf	Rib
Jurk	Kleid	Piek	Gipfel	Rit
Kast	Schrank	Poes	Katze	Rug
Kerk	Kirche	Pols	Puls	Rups
Kier	Spalt	Pont	Faehre	Schok
Kip	Huhn	Prik	Spritze	Sla
Klant	Kunde	Rek	Regal	Stof
Koor	Chor	Rij	Reihe	Tas
Kop	Tasse	Rijst	Reis	Trui
Kras	Kratzer	Roer	Ruder	Tuin
Kruk	Kruecke	Rouw	Trauer	Vork
Krul	Locke	Rust	Ruhe	Vorst
Kust	Kueste	Schol	Scholle	Vos
Kwal	Qualle	Sjaal	Schal	Walm
Lens	Linse	Slot	Schloss	Wol
Lijm	Kleber	Snor	Schnurbart	Zalm
Loof	Laub	Som	Summe	Zeil
Luis	Laus	Spaak	Speiche	

Melk	Milch	Spier	Muskel
Mond	Mund	Spoor	Gleis
Neus	Nase	Stoot	Stoss
Nier	Niere	Stuur	Lenkrad
Pijp	Pfeife	Taal	Sprache
Plak	Scheibe	Tand	Zahn
Raam	Fenster	Teef	Zecke
Rem	Bremse	Teek	Zecke
Reu	Ruede	Tent	Zelt
Riem	Leine	Trap	Treppe
Schat	Liebling	Vacht	Fell
Sluis	Schleuse	Veer	Feder
Soep	Suppe	Vent	Kerl
Tijd	Zeit	Vis	Fisch
Tong	Zunge	Vuil	Schmutz
Uur	Stunde	Vuur	Feuer
Vlees	Fleisch	Wei	Wiese
Waard	Wirt	Wijk	Stadtteil
Wet	Gesetz	Will	Wille
Winst	Gewinn	Wond	Wunde
Worst	Wurst	Zeep	Seife
Zalf	Salbe	Zin	Sinn
Zout	Salz	Zuil	Saeule
Sap	Saft	Steen	Stein
Zon	Sonne	Taart	Kuchen

Figure S1: **Models simulated with ROAST toolbox in Matlab**, showing the electrical fields expected with the stimulation. Red electrode pad represents the target electrode, blue represents the return electrode.

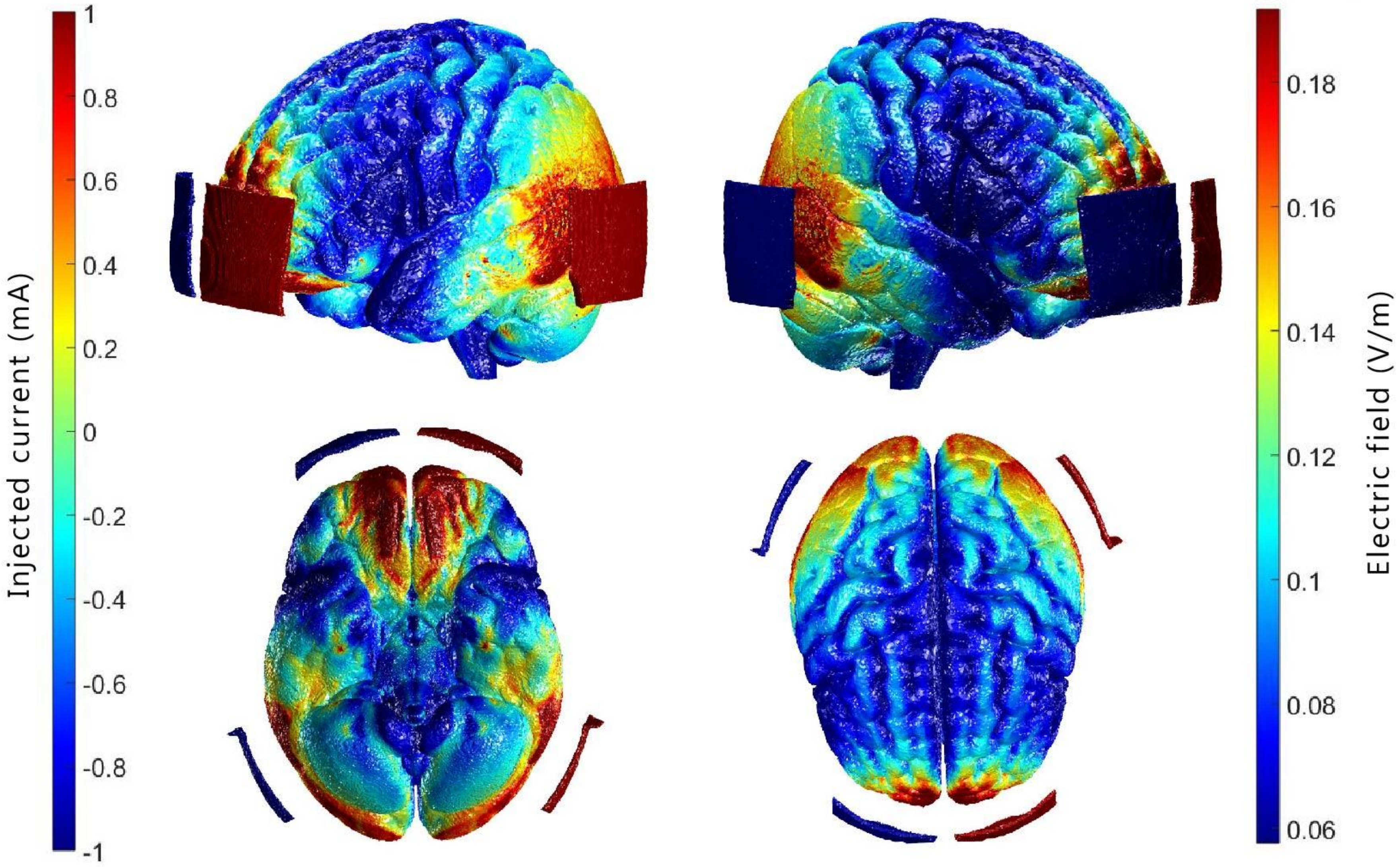

Table S2: **Values and analyses of objective sleep parameters and oscillatory power during sleep**.

	**Continuous**	**Time-locked**	***t***	***p***	***d***
**Objective sleep parameters** (N=36)					
Total Time in Bed	489 ± 6.61	520 ± 3.78	-2.93	**.006****	0.91
WASO [%]	6.28 ± 0.77	3.65 ± 0.37	2.26	**.031***	0.69
N1 [%]	5.14 ± 0.54	4.75 ± 0.45	0.39	.697	0.13
N2 [%]	44.53 ± 1.19	45.90 ± 1.03	-0.61	.548	0.20
N3 [%]	12.80 ± 0.64	16.49 ± 0.90	-2.22	**.036***	0.80
REM [%]	20.98 ± 0.85	20.68 ± 0.54	0.21	.832	0.07
WASO [min]	31.24 ± 3.94	19.23 ± 1.95	1.99	.055[a]	0.61
N1 [min]	25.64 ± 2.82	24.90 ± 2.49	0.14	.892	0.05
N2 [min]	217.45 ± 5.95	237.37 ± 4.66	-1.85	.073[a]	0.60
N3 [min]	62.55 ± 3.08	85.43 ± 4.79	-2.66	**.015***	0.97
REM [min]	102.95 ± 4.09	107.20 ± 2.89	-0.60	.552	0.19
TST [min]	408.56 ± 6.86	454.90 ± 3.40	-4.42	**<.001*****	1.35
SOL [min]	17.02 ± 2.25	24.27 ± 2.96	-1.30	.205	0.46
Stimulation time [%]	10.26 ± 0.36	8.51 ± 0.19	3.18	**.003****	0.98
Stimulation time [min]	49.69 ± 1.37	44.03 ± 0.91	2.46	**.019***	0.78
SWS latency [min]	30.0 ± 4.51	31.10 ± 5.91	-0.10	.922	0.04
REM latency [min]	101.17 ± 5.49	99.23 ± 4.29	0.20	0.85	0.06
**Oscillatory Power [µV] during sleep** in the whole night, frontal region (N=36)					
Beta	0.027 ± 0.005	0.023 ± 0.002	0.98	.336	0.31
Theta	0.820 ± 0.073	1.193 ± 0.114	-2.67	**.014***	0.96
SWAB/B	543.93 ± 47.13	738.58 ± 48.34	-2.47	**.021***	0.87
**Oscillatory Power [µV] during sleep** in the first cycle, frontal region (N=37)					
Beta	0.017 ± 0.001	0.027 ± 0.003	-2.25	**.036***	0.83
Theta	1.188 ± 0.116	1.700 ± 0.153	-2.66	**.012***	0.90
SWA/B	1125.40 ± 96.03	1305.53 ± 113.10	-0.93	.359	0.31
**Oscillatory Power [µV] during sleep**					

in the second cycle, frontal region (N=37)					
Beta	0.018 ± 0.002	0.022 ± 0.002	-1.29	.208	0.42
Theta	0.895 ± 0.087	1.295 ± 0.131	-2.40	**.024***	0.83
SWA/B	892.02 ± 92.24	928.18 ± 87.43	-0.22	.826	0.07

Notes: Objective sleep values are based on polysomnographic recordings. Non-rapid eye movement sleep (NREM) stage 1, 2, 3 (N1, N2, N3), rapid eye movement sleep (REM), Wake after sleep onset (WASO), total sleep time (TST), sleep onset latency (SOL), slow wave sleep (SWS) and REM latency are measured in minutes [min] and percentages indicated parietal percentage of TST [%]. For one participant of the *time-locked* group, the EEG sleep data was not recorded for the whole night, therefore, the analyses on general sleep parameters were performed on 36 participants. The oscillatory power was calculated on the complete night, and separately for the first two sleep cycles. The values are reported in microvolts. Values are Means (M) ± Standard Error of the Mean (SEM). * indicates p < .05, ** indicates p ≤ .01, *** indicates p ≤ .001, [a] indicates p ≤ .09. Significant results are highlighted in bold. *d* represents the effect sizes where a value below or equal to 0.2 reflects a small effect, a value between 0.2 and 0.8 reflects a medium effect, and a value above 0.8 reflects a large effect.

Table S3: Number of Gains and Losses in *continuous* and *time-locked* group for each stimulation condition (theta-tACS, control-tACS and uncued).

	Continuous			Time-locked			Main effect of stimulation			Interaction effect		
	**Theta-tACS**	**Control-tACS**	**Uncued**	**Theta-tACS**	**Control-tACS**	**Uncued**	**F**	***p***	**η^2**	**F**	***p***	**η^2**
**Gains**	1.76 ± 0.24	1.90 ± 0.27	2.62 ± 0.25	1.88 ± 0.27	2.13 ± 0.24	2.25 ± 0.15	1.81	.171	.05	0.39	.676	.01
**Losses**	2.95 ± 0.29	2.33 ± 0.25	3.05 ± 0.38	3.31 ± 0.34	3.81 ± 0.30	3.25 ± 0.29	0.09	.916	<.01	1.38	.257	.04

Notes: Gains represents the items that were not correctly remembered before sleep but correctly remembered after sleep. Losses represent the items that were correctly remembered before sleep but not correctly remembered after sleep. Values are Means (M) ± Standard Error of Mean (SEM). η^2 represents the effect sizes where a value below or equal to .02 reflects a small effect, a value between .02 and .14 reflects a medium effect, and a value above .14 represents a large effect.

Figure S2: **Number of A) Gains and B) Losses** in *continuous* and *time-locked* group for each stimulation condition (theta-tACS, control-tACS and uncued. For both categories (gains and losses), no differences was found between the different stimulation conditions (both *p*-values > .171), nor between the two groups (both *p*-values > .096). No interaction was found (both *p*-values > .257).

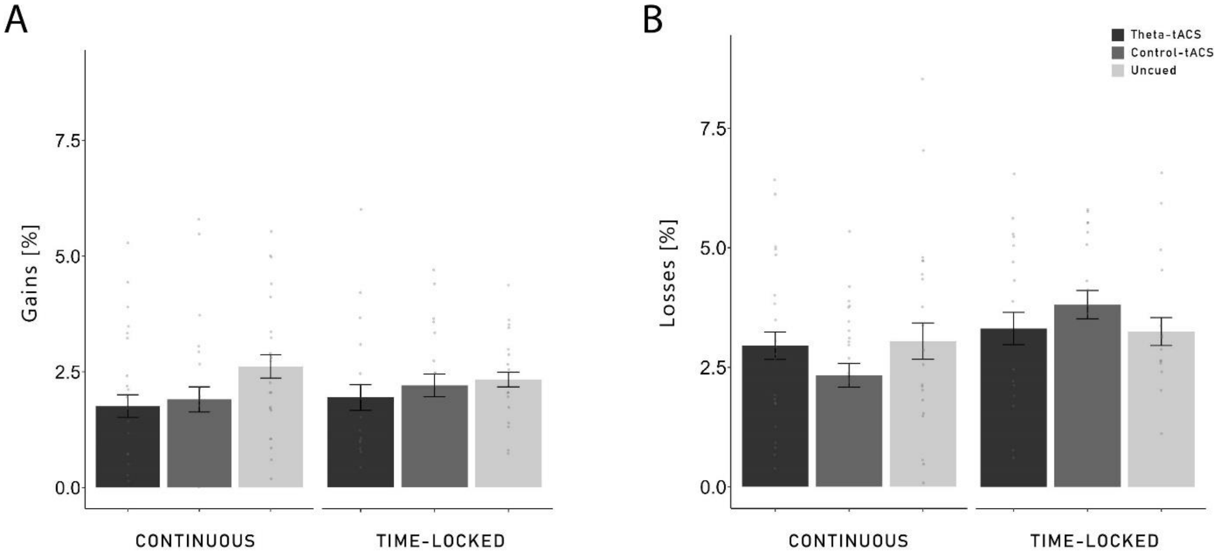

Figure S3: **Average oscillatory power** during the learning phase (pre-sleep recall) of the *time-locked* group, recorded in the frontal channels (F3, F4, F7, F8, Fz, FC5, FC6). **A) Oscillatory power changes for all words** (subsequently remembered, subsequently forgotten) during the learning phase. An increase in theta power (4-7 Hz) was observed 0.54 – 1 sec after cue onset for all words (p = .002). **B) However, no differences in theta power were observed when comparing subsequently remembered words and subsequently forgotten words**.

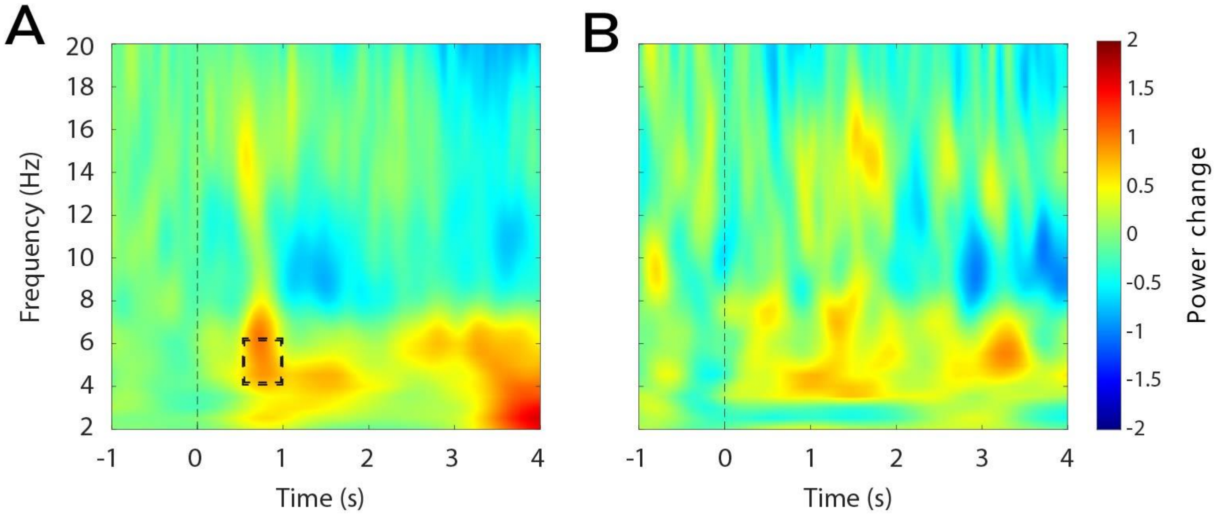

Supplement: Supplementary file 1 [file clockssleep-06-00015-s001.zip › clockssleep-2846405-supplementary.pdf]
